# Common Variants on Chromosome 9p21 Are Associated with Normal Tension Glaucoma

**DOI:** 10.1371/journal.pone.0040107

**Published:** 2012-07-05

**Authors:** Mitsuko Takamoto, Toshikatsu Kaburaki, Akihiko Mabuchi, Makoto Araie, Shiro Amano, Makoto Aihara, Atsuo Tomidokoro, Aiko Iwase, Fumihiko Mabuchi, Kenji Kashiwagi, Shiroaki Shirato, Noriko Yasuda, Hidetoshi Kawashima, Fumiko Nakajima, Jiro Numaga, Yoshiya Kawamura, Tsukasa Sasaki, Katsushi Tokunaga

**Affiliations:** 1 Department of Ophthalmology, Graduate School of Medicine, The University of Tokyo, Tokyo, Japan; 2 Department of Human Genetics, Graduate School of Medicine, The University of Tokyo, Tokyo, Japan; 3 Department of Ophthalmology, Tajimi Municipal Hospital, Gifu, Japan; 4 Tajimi Iwase Eye Clinic, Gifu, Japan; 5 Department of Ophthalmology, University of Yamanashi, Yamanashi, Japan; 6 Shirato Eye Clinic, Tokyo, Japan; 7 Department of Ophthalmology, Tokyo Metropolitan Police Hospital, Tokyo, Japan; 8 Department of Ophthalmology, Saitama Red Cross Hospital, Saitama, Japan; 9 Department of Ophthalmology, Showa General Hospital, Tokyo, Japan; 10 Department of Ophthalmology, Tokyo Metropolitan Geriatric Hospital, Tokyo, Japan; 11 Department of Psychiatry, Yokohama Clinic, Kanagawa, Japan; 12 Department of Physical and Health Education, Graduate School of Education, The University of Tokyo, Tokyo, Japan; Centre for Eye Research, Australia

## Abstract

Although intraocular pressure (IOP) is the most definitive cause of glaucoma, a subtype of open angle glaucoma (OAG) termed normal tension glaucoma (NTG), which occurs in spite of normal IOP, accounts for a large part of glaucoma cases, especially in Japan. To find common genetic variants contributing to NTG in Japanese patients, we conducted a genome-wide association study (GWAS). We performed the first screening for 531,009 autosomal SNPs with a discovery cohort of 286 cases and 557 controls, and then a second screening for the top 30 suggestive loci in an independent cohort of 183 cases and 514 controls. Our findings identified a significantly associated SNP; rs523096 [combined p-value = 7.40× 10^−8^, odds ratio (OR)  = 2.00 with 95% confidence interval (CI) 1.55–2.58] located 10 kbp upstream of *CDKN2B* on chromosome *9p21*. Moreover, analysis of another independent case-control set successfully replicated the results of the screening studies (combined values of all 3 stages p = 4.96 × 10^−11^, OR  = 2.13 with 95% CI 1.69–2.68). The SNPs near rs523096 were recently reported to be associated with OAG associated with elevated IOP in primary open-angle glaucoma (POAG), the predominant subtype of glaucoma in Caucasian populations. Our results revealed that the *9p21* locus is also associated with NTG in Japanese. In addition, we identified SNPs more strongly associated with NTG.

## Introduction

Glaucoma is a neurodegenerative disease that leads to progressive loss of retinal ganglion cells [1], causing irreversible visual field defects [2], and one of the leading causes of irreversible blindness, affecting over 60 million people worldwide [3]. Presently, glaucoma is clinically defined by the existence of characteristic changes in the optic disc and corresponding visual field defects [4]. Elevation of intraocular pressure (IOP) is the only proven cause of glaucoma to date and lowering IOP is the only established therapy for reducing the rate of deterioration of the visual field in glaucoma patients [5]–[8].

Open-angle glaucoma (OAG) is the most prevalent type of glaucoma, and characterized by adult onset and chronic IOP-dependent progression. OAG associated with IOP consistently within a statistically normal range (≤21 mmHg) has been termed normal-tension glaucoma (NTG) [4].

NTG is thought to be included in the category of OAG. However, in addition to the obvious lower IOP than OAG in a classical sense, OAG is also associated with elevated IOP (≥22 mmHg) and primary open angle glaucoma (POAG), with several differences between NTG and POAG reported. Optic disc hemorrhage, an important and well-established negative prognostic factor for glaucoma, is much more frequently seen in NTG [9]–[12]. The majority of reported studies have noted small, but significant differences in the patterns of damage in the optic disc, retinal nerve fiber layer, and visual field [13]–[21]. Furthermore, immune aberrations [22]–[26], and abnormalities in systemic [27]–[31] and/or ocular circulation [32]–[34] are reportedly more likely to be encountered in NTG than POAG. Although the exact etiology of glaucomatous optic neuropathy is unknown, neuronal factors independent of IOP or factors to define hypersensitivity to IOP rather than elevating IOP have been suggested to underlie the etiology of NTG. Interestingly, there are differences among affected populations regarding the prevalence of NTG. In Caucasians and African-Americans, the prevalence ratios of NTG and POAG are approximately 1.0 [35]–[39], while those are about 3 in Koreans [40] and greater than 10 in Japanese [41].

Although familial aggregation of POAG has been reported [42], that has not been well investigated for NTG. Linkage analysis of a British family with NTG revealed *optineurin* as a gene causative of NTG [43], [44]. However, that mutation is very rare in other populations including Japanese [45]–[47]. In other candidate gene approaches, possible associations of *apolipoprotein E* [48]–[50], *OPA1* [51]–[53], and *toll-like receptor 4* [54] with NTG have been investigated, though the results were inconsistent. Recently, a genome-wide association study (GWAS) of 305 Japanese NTG patients and 355 controls revealed that rs3213787 in *S1 RNA binding domain* on chromosome 2 was significantly associated with NTG [55], [56].

Two GWASs for POAG in Caucasians have been reported, in which 3 genetic loci, *caveolin 1* (*CAV1*) and *caveolin 2* (*CAV2*) on *7q31* [57], *TMCO1* on*1q24*, and *cyclin-dependent kinase inhibitor 2B antisense RNA* (*CDKN2BAS*) on *9p21* [58], were identified as susceptibility loci, while another GWAS of Japanese POAG patients provided only suggestive results [59].

In an attempt to identify genetic factors contributing to NTG, we conducted a GWAS for NTG in Japan, where a high prevalence rate of NTG has been observed. In the present study, a total of 620 definitively diagnosed NTG patients and 1258 controls were investigated. After GWAS in our discovery cohort, a second screen was performed for the top 30 suggestive loci. Thereafter, replication analysis was performed for an SNP found to be significantly associated in the first and second screening sets.

## Results

### First and Second Screenings, and Replication Study

**Figure 1:**
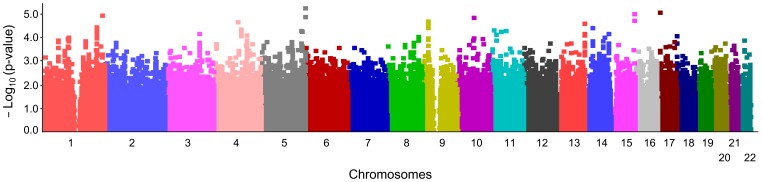
Manhattan plots of genome-wide association study for NTG. Negative common logarithm of p-value plots for genome-wide association study in the first screening. The X-axis indicates chromosomes and their positions, and the Y-axis indicates the negative common logarithm of the association p-value of 531,009 SNPs. No SNP showed a genome-wide significance at p = 9.416 × 10^−8^ (Bonferroni’s correction).

In the first screening, we performed a GWAS using discovery cohort of 286 cases and 557 controls, each of which had passed the sample quality control (QC) criteria. We applied an SNP QC [minor allele frequency (MAF) ≥0.05, call rates ≥0.98, Hardy-Weinberg equilibrium p-value ≥0.001 in controls, and visual cluster removal] and selected 531,009 SNPs for the first screening. We generated a quantile-quantile plot to inspect possible population stratification effects and obtained the genomic inflation factor (λ) of 1.046, indicating no population substructure (Figure S1). However, none of the SNPs reached genome-wide significance in the first screening. The resulting Manhattan plot is shown in Figure 1.

To identify significantly associated SNPs, we carried out the second screening using an independent set of 183 NTG cases and 514 controls. We genotyped 30 SNPs on the top 30 loci shown to be the most significant in the first screening. The results of association analysis in the second screening are shown in Table S2. We observed a significant association at a single SNP, rs523096 on chromosome *9p21* (p = 4.55 × 10^–4^, OR  = 2.15 with 95% CI 1.39–3.32 in the second screening). The combined results of the first and 2nd screenings showed genome-wide significance (rs523096, combined p = 7.40×10^–8^, OR  = 2.00 with 95% CI 1.55–2.58).

In the replication study, we performed genotyping of rs523096 for the replication cohort composed of 151 cases and 187 controls independent of the first and second screening, and the same significant association was obtained (p = 1.63 × 10^–4^). Thus, we confirmed the association of chromosome *9p21* with NTG. Finally, the combined p-value of all 3 stages was 4.96× 10^–11^ (OR  = 2.13 with 95% CI 1.69–2.68), which indicated a definite association (Table 1).

**Table 1:** Results of rs523096 in each case-control set.

	Frequency of A allele				
	Control	Case	HW-p in control	P-value	OR(95% CI)
**Discovery cohort (First screening)**	0.825	0.904	0.018	1.59 × 10^−5^	1.99(1.45–2.74)
**Second screening**	0.859	0.929	0.86	4.55 × 10^−4^	2.15(1.39–3.32)
First and second screening sets^a^				7.40× 10^−8^	2.00(1.55–2.58)
**Replication cohort**	0.840	0.934	0.79	1.63 × 10^−4^	2.69(1.58–4.58)
All 3 case-control sets^b^				4.9 6× 10^−11^	2.13(1.69–2.68)

Results of association analysis of rs523096 in discovery cohort and second screening sets, and replication cohort.

a p-value and odds ratio for combined data from first and second screening set.

b p-value and odds ratio for combined data of all 3 case-control sets.

HW-p: p-value for Hardy-Weinberg equilibrium, OR: odds ratio, 95% CI: 95% confidence interval.

### Dense Association Mapping of *9p21*

**Figure 2:**
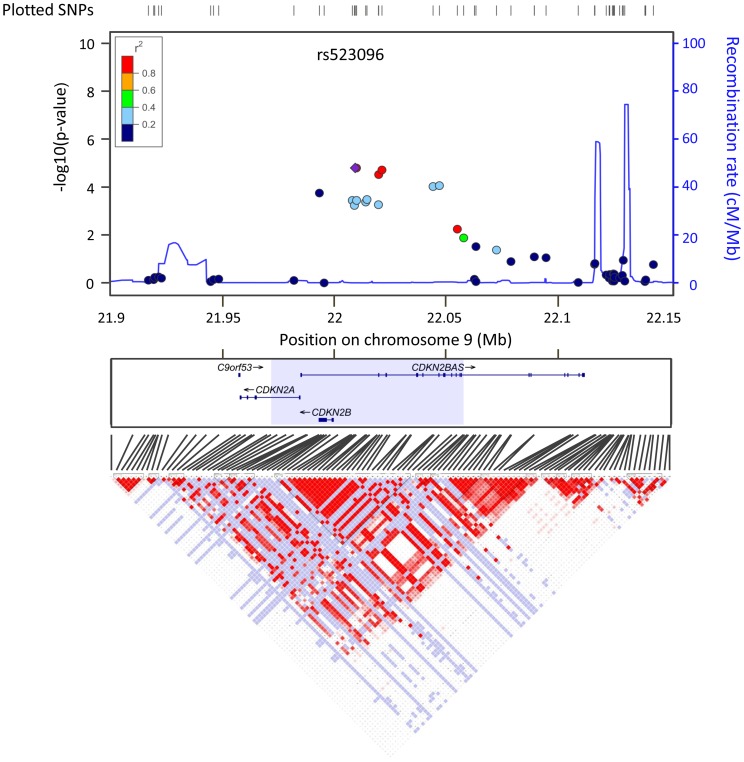
LD plots on *9p21* in first screening data in dense association mapping region. Upper panel shows plots of negative common logarithm of association p-values for *9p21* locus. Each dot represents an SNP from the first screening and the colors of the dots represent the extent of linkage disequilibrium with rs523096 (purple diamond). Shaded area in the middle panel of the gene structures indicates the 87-kbp region, defined as the dense association mapping region. Lower panel shows an LD plot (D-prime) of Japanese specimens from HapMap III data.

We further analyzed SNPs on chromosome *9p21* using the second screening case-control samples in dense association mapping. The 87-kbp region for dense association mapping was defined to cover the strong linkage disequilibrium (LD) region, including the associated SNPs in the first screening (Figure 2). The association plots on *9p21* in the first screening data, the region for the dense association mapping, and the LD plot calculated from the Japanese specimens of HapMap III using Haploview 4.2 are shown in Figure 2. A total of 29 SNPs (28 SNPs newly genotyped and rs523096) were entered into the analysis (Table S3). The association was reproduced in SNPs with a high LD with rs523096 in the second screening set. In dense association mapping, rs643319 showed the lowest p-value (p = 2.57× 10^−5^, OR  = 1.78 with 95% CI 1.36–2.33). In addition, 10 SNPs around these 2 SNPs showed p-values lower than or nearly equal to that of rs523096. Combined with the results of the first GWAS data set, a total of 6 SNPs including the representative SNP rs523096 showed p-values markedly below the genome-wide significance of p = 9.416 × 10^−8^ (Table S3). Imputation analysis in the region showed no evidence of SNPs with a p-value extremely lower than those of rs523096, rs643319, or other SNPs (Figure S2).

The results of haplotype analysis are shown in Table 2. One haplotype carrying the G allele of rs523096 was most significantly associated and shown to be protective against the disease.

**Table 2:** Haplotype analysis of dense association mapping region.

										Frequency		
No.	Haplotypes									Case	Control	p-value
**Block 1**	rs3731217	rs2811710										
haplotype 1	A	C								0.743	0.717	0.341
haplotype 2	C	T								0.172	0.208	0.144
haplotype 3	A	T								0.085	0.075	0.558
**Block 2**	rs3217992	rs1063192	rs2285329	rs3808845	rs643319	rs523096	rs10738604	rs2151280	rs12352425			
haplotype 1	T	A	A	G	C	A	A	A	G	0.540	0.440	0.00110
haplotype 2	C	A	A	A	A	A	G	G	G	0.161	0.208	0.0562
haplotype 3	C	G	A	G	A	G	G	G	G	0.063	0.132	4.00 × 10^−4^
haplotype 4	C	G	A	G	C	A	A	A	G	0.082	0.061	0.170
haplotype 5	C	A	A	G	C	A	G	A	A	0.055	0.054	0.983
haplotype 6	T	A	G	G	C	A	A	A	G	0.041	0.059	0.204
haplotype 7	T	A	A	G	C	A	A	G	G	0.026	0.014	0.0925
**Block 3**	rs8181047	rs10965224										
haplotype 1	G	A								0.792	0.725	0.0110
haplotype 2	A	T								0.107	0.176	0.00180
haplotype 3	G	T								0.101	0.100	0.935

Haplotypes with frequencies >0.01 are shown. Each character indicates each allele in the forward strand of human genome build 36.

These results showed that multiple SNPs in this region are strongly associated with NTG.

### Evaluation of POAG-associated SNPs

Results of the first screening of SNPs alternative for POAG-associated SNPs (rs17588172 for rs1052990, and rs6969706 for rs4236601 of the *CAV1* and *CAV2* regions on *7q31* [57], and rs4657477 for rs4656461 and rs7518099 of the*TMCO1* region on *1q24* [58]) are shown in Table S4. The frequencies of the alleles are also shown, which were consistent with those reported as risk alleles for POAG [57], [58]. We were unable to obtain replicated results for these 3 SNPs, though the minor allele frequencies of rs4657477 and rs6969706 were so low that evaluation of an association was difficult. In addition, the effects of both rs4657477 on *1q24* and rs17588172 on *7q31* appeared to be opposite, as compared to results presented in previous reports.

The association between 6 SNPs (rs547984, rs540782, rs693421, rs2499601, rs7081455, and rs7961953) and Japanese POAG suggested in the previous GWAS [59] was not demonstrated in our first screening data (Table S5). The statistical powers for these SNPs indicated sufficient values (0.789 to 0.933, Table S5).

## Discussion

In the present study, we found a significant association of rs523096, which is located 10 kbp upstream of *cyclin-dependent kinase inhibitor 2B* (*CDKN2B*) and in the intron of *CDKN2BAS* (also known as *ANRIL*) on *9p21*, with Japanese NTG. This locus is one of 3 genetic loci recently reported to be associated with POAG in Caucasian GWASs. In one of those studies, rs4977756 located 60 kbp upstream of *CDKN2B*, was most significantly associated [58]. Although rs4977756 was not included in our analysis, rs10965224, which was shown to be in absolute LD with rs4977756 (r^2^ = 1 in Japanese in Tokyo (JPT) of HapMap III), exhibited a relatively weak association (p = 0.013, OR  = 1.45 in discovery cohort, Table S3). Two other groups have reported an association between Caucasian POAG and rs1063192 in the 3′ UTR of *CDKN2B* [60], [61]. Rs1063192 was originally reported as a lead SNP in a GWAS of normal variations of vertical cup to disc ratio (VCDR) in a general population [62]. In our data obtained with dense association mapping, the p-value for rs1063192 was 0.038, with an OR of 1.41. For these 2 SNPs, our results for Japanese NTG were similar in regard to the ORs of those for Caucasian POAG cohorts (1.39 for rs4977756 [58], and 1.32 and 1.37 for rs1063192 [60], [61]). Rs523096, which had an association identified in the present study, and the SNPs in absolute LD (rs2069418, rs573687, rs518394, rs564398, and rs7865618) in Japanese HapMap specimens showed high ORs (highest = 2.20 with 95% CI 1.40–3.46 for rs2069418) in dense association mapping. We identified multiple SNPs with more significant associations as compared to SNPs previously reported in Caucasians, though we were unable to identify a single SNP as the primary variant of this region using haplotype and imputation analyses. Furthermore, a high frequency of the risk allele of rs523096 (or low frequency of the protective allele) in Japanese as compared to that in Caucasians (0.907 vs. 0.551 in HapMap data) may be an explanation for the high prevalence rate of NTG in Japanese [41].

Consistently, a very recent report on the study of Japanese NTG by other independent GWAS group demonstrated rs523096, rs518394, rs564398 and rs7865618 were significant association SNPs with NTG and rs523096 was the most significant one [63]. In our study, selection criteria were more restricted to select severer and younger patients, and combining with all stages, stronger association of rs523096 with NTG was observed, and more SNPs on the *CDKN2BAS* region were demonstrated as genome-wide significance and some were more significant than reported SNPs [rs643319(p = 5.44×10^−8^), rs10965219(p = 2.38×10^−8^), rs10120688(p = 3.57×10^−8^)].

*Cyclin-dependent kinase inhibitor 2A* (*CDKN2A*) encoding p16^INK4a^ and p14^ARF^, *CDKN2B* encoding p15^INK4b^, and non-protein coding *CDKN2BAS* are located on the *9p21* locus. The cyclin-dependent kinase inhibitors p16^INK4a^ and p15^INK4b^ function as cell growth regulators to control cell cycle G1 progression, and are well-known tumor suppressor genes [64]. Germline mutations on *9p21* have been reported to be associated with familial predisposition to melanoma [65] and homozygous deletion on *9p21* is frequently detected in a wide variety of tumors [66]. Also, the expression of *CDKN2B* was found to be dramatically induced by transforming growth factor beta (TGF-β) [67], suggesting its role in TGF-β induced growth inhibition. TGF-β is also known to be involved in programmed cell death in the developing retina and optic nerve [68], [69], and has also been suggested to play an important role in glaucoma [69]–[71]. In fact, in the region of associated SNPs, a regulatory domain interacting with TGF-β-mediated proteins (Smad, sp1, miz-1, and myc) has been reported [72], [73] and rs2069418, which showed the highest OR in dense association mapping, is located in the Smad binding region [72], [73]. The products of *CDKN2A* regulate the Rb and p53 pathways, which induce cell cycle arrest, apoptosis, and senescence [74], and are reported to be involved in retinal ganglion cell apoptosis [75]. *CDKN2BAS* is a large antisense non-coding gene that overlays the *CDKN2B* gene in an antisense strand. Although the function of *CDKN2BAS* is not well elucidated, recent GWASs of several common diseases (coronary artery disease [76], type 2 diabetes [76], aortic aneurysm [77], intra-cranial aneurysm [76], endometriosis [78], glioma [79], [80]) have revealed associations with this long non-coding region. Also, *CDKN2BAS* has been suggested to influence the expressions of *CDKN2B* and *CDKN2A* [81], [82].

Measurement of IOP using Goldmann applanation tonometery is known to be influenced by central corneal thickness (CCT) with a thinner CCT giving a lower value [83]. In the present study, diagnosis of NTG was based on IOP≤21 mmHg without regard to CCT and we can not rule out the possibility that POAG eyes with thin CCT were misclassified as NTG. However, it was also reported that CCT in Japanese patients with NTG showed no significant difference from that in POAG patients or normal subjects [41], [84], thus we believe that such misclassification occurred in an extremely small portion of our cases.

In conclusion, we identified a susceptibility locus related to Japanese NTG. The locus on *9p21* is considered to be common in Caucasian POAG and Japanese NTG patients, while the SNPs on this locus showed stronger associations with Japanese NTG than with previously reported POAG-associated SNPs in Caucasians. Considering the population diversity of prevalence rates and sensitivity to IOP, our results are important for elucidating the pathogenesis of OAG, and support a hypothesis stating that the effect of 9p21 is responsible for the neuronal factor rather than high IOP in glaucomatous optic neuropathy, since a common genetic factor may be involved irrespective of baseline IOP. Further investigation is needed to identify the disease causative variant and reveal how genes on the locus play a role in the etiology of glaucomatous optic neuropathy.

## Materials and Methods

### Ethics Statement

The study protocol was approved by the Research Ethics Committees of Graduate School of Medicine, The University of Tokyo, and all participants provided written informed consent after an explanation of the nature and possible consequences of the study.

### Subjects

All participants in the present study were of Japanese ancestry. A total of 620 NTG patients registered at The University of Tokyo Hospital, Tajimi Municipal Hospital, Iwase Tajimi Clinic, Yamanashi University Hospital, Shirato Eye Clinic, Tokyo Metropolitan Police Hospital, Saitama Red Cross hospital, or Showa General Hospital were enrolled.

All affected subjects fulfilled the following diagnostic criteria: (1) existence of glaucomatous optic disc change, judged by glaucoma specialists, (2) existence of visual field loss consistent with a neuronal lesion [visual field loss evaluated using a Humphrey visual field analyzer (Zeiss-Humphrey) central 30–2 full threshold, Swedish Interactive Thresholding Algorithm Standard (SITA-S) Central 30-2 program, or SITA-S 24-2, according to Anderson and Patella’s criteria [85]], (3) untreated IOP with a level consistently ≤21 mmHg during the follow-up period, (4) spherical equivalent refraction between −8.0 and 5.0 diopter, and (5) no apparent neurosurgical or otorhinological abnormalities that might cause glaucomatous changes in the optic disc or visual field defect. In addition the selection criteria were modified based on subject age, as follows: (1) no modification if the patient was diagnosed below the age of 50 years, (2) –5.00 dB of or worse in mean deviation measured by Humphrey visual field analyzer in at least one eye if the disease was diagnosed when the patient was between the ages of 50 and 65 years, (3) −10.00 dB or worse in at least one eye if the disease was diagnosed when the patient was older than 65 years. We divided these subjects into 3 stages (first screening, second screening, replication study). The clinical manifestations of the NTG cases in each stage are shown in Table S1.

Control samples for the first screening were comprised of 578 Japanese healthy volunteers from the Departments of Human Genetics [86] and Neuropsychiatry [87] of The University of Tokyo Hospital. Their data were obtained using the CEL files of Affymetrix Genome-Wide Human SNP array 6.0. Of these, 557 samples fulfilled the sample QC threshold described below and were used for association analysis. Control samples in the second screening and replication study were healthy volunteers from Tajimi Municipal Hospital and the Department of Human Genetics, The University of Tokyo. Control subjects did not undergo an ophthalmic examination. All DNA samples used in this study were derived from individuals after they had given written informed consent.

### SNP Genotyping and Data Cleaning in First Screening

In the first screening, we genotyped 310 Japanese patients with NTG using Affymetrix Genome-Wide Human SNP Array 6.0 (Affymetrix, Santa Clara, CA), according to the manufacturer's instructions. After exclusion of 24 cases and 19 control samples with QC call rates <95%, contrast QC <0.4, or a difference of contrast QC of Sty and that of Nsp>2, the remaining 286 case samples and 558 control samples were recalled using Birdseed version 2 software (Affymetrix). Next, identity by descent (IBD) was estimated using plink v1.07 [88] and 1 of a pair of control samples with pi-hat >0.125 was excluded from the first screening set. The pi-hat values for the other pairs were <0.125, showing no sample duplication or existence of unexpected relatives.

We applied the following thresholds for QC in data cleaning: Hardy-Weinberg equilibrium with a p-value ≥0.001 for control samples, call rate for each SNP≥0.98, and MAF ≥0.05. All cluster plots for the SNPs showing a p-value <0.001 in association analyses were checked by visual inspection. SNPs with ambiguous genotype calls were excluded. A total of 531,009 SNPs on autosomal chromosomes passed the QC filters and were subjected to association analysis.

Although the 24 case samples noted above were excluded from the first screening by data cleaning, the quality of their DNA was adequate for SNP typing in the second screening and they were included in that screening.

### Second Screening

The case and control sets for the second screening included 183 cases and 514 controls. To identify associated variants with high odds ratios (ORs), 30 SNPs were selected from the top list in the first screening.

Thirty SNPs were genotyped using the DigiTag2 method [89] and SNPs with a call rate >0.97 were adopted. Three of these 30 SNPs that could not pass the filter were genotyped again using a TaqMan assay (Applied Biosystems, Carlsbad, CA). The average call rate of the 30 SNPs was 0.997.

A DigiTag2 assay was performed as described by Nishida *et al* [89]. This assay enables determination of 96 or 32 SNPs genotypes at once. A multiplexed oligonucleotide ligation assay was performed after multiplex PCR, and a labeling reaction was achieved with two 5′ query probes and 1 common probe prepared for a single SNP site. The 5′ query probes had a sequence complementary to that of the 5′ sequence flanking the target SNP and each of the probes had an allele-specific sequence. Following this, a hybridization reaction was performed using a DNA microarray (NGK Insulators, Nagoya, Japan) with separated areas containing oligonucleotide probes for the SNPs. The genotype calls were determined using SNPStar software (version 0.0.0.8, Olympus, Tokyo, Japan) [89].

TaqMan assays were performed using a LightCycler 480 System II (Roche, Bazel, Switzerland), according to the manufacturer’s protocol.

### Replication Study

An independent set of 151 cases and 187 controls was included in the replication study. Rs523096, the significantly associated SNP in the screening studies, was genotyped using a TaqMan assay.

### Dense Association Mapping of *9p21*

For dense association mapping of *9p21*, 33 SNPs on the 87-kbp region of *9p21* were planned to be newly genotyped for the second screening case-control set in addition to rs523096. To cover the strong LD region where associated SNPs in the first screening were distributed (53-kbp region between rs3217992 and rs10120688), the 87-kbp region was defined as the dense association mapping region.

In the 87-kbp region of dense association mapping, we extracted SNPs with an MAF >0.01 from Japanese specimens of HapMap phase III. SNPs in absolute LD (r^2^ = 1) with already selected SNPs were omitted, except for those with rs523096. All SNPs in absolute LD with rs523096 were included. In addition, an SNP previously reported to strongly affect *CDKN2B* expression [90] and 6 SNPs located just upstream of *CDKN2B* were selected. Of 33 SNPs, 32 were genotyped using the DigiTag2 method and 23 with call rates >0.97 were adopted. Four of 9 SNPs failed to be genotyped in DigiTag2 assays, while rs2069426 was genotyped using a TaqMan Assay. Finally, 29 SNPs were entered into the analysis. The average call rate of the 29 SNPs was 0.996 and Hardy-Weinberg equilibrium p-values for the controls were >0.05 for all adopted SNPs.

### Evaluation of POAG-associated SNPs

We evaluated associations between NTG and SNPs on the other 2 loci found to be associated with POAG in previous GWASs [57], [58], as well as SNPs whose associations were suggested in the GWAS of Japanese POAG [59] using data obtained in our first screening.

The POAG-associated SNPs of rs1052990 and rs4236601 on *7q31* [57], and rs4656461 and rs7518099 on *1q24* [58] were not included in our first screening data, thus we extracted alternative SNPs that were in absolute LD (r^2^ = 1) with them in HapMap data from the SNPs in the first screening. As a result, rs17588172 for rs1052990, rs6969706 for rs4236601, and rs4657477 for rs4656461 and rs7518099 were selected as alternative SNPs. Because of the absence of polymorphism at rs4236601 in JPT of HapMap III, only rs4730748 was selected according to the LD in Chinese in Beijing (CHB).

Six SNPs (rs547984, rs540782, rs693421, rs2499601, rs7081455, and rs7961953) reported in a GWAS of Japanese POAG [59] were directly evaluated in our first screening data. For these, statistical powers were calculated after applying minor allele frequencies in our control samples for risk allele frequencies, odds ratios for reported ones [59], and 0.05 for alpha.

### Statistical Analysis

In the genome-wide association analyses of the first and second screenings, as well as replication analysis we evaluated the association of each SNP using an allelic test. In the first screening, to ascertain whether the control samples were properly matched to the cases, the genomic inflation factor (λ) was estimated using the median chi-square value from the allelic test. The combined p-values of individual case-control sets were calculated based on the simply combined datasets. We set p = 1.667 × 10^−3^ as the significant p-value in the second screening only and p = 9.416 × 10^−8^ as the genome-wide significant value, according to Bonferroni’s correction. Deviations from Hardy-Weinberg equilibrium were calculated using exact test. [91].

Genotype data from Japanese specimens of HapMap Phase II were used as reference panels in imputation analysis. Association and imputation analyses were performed as implemented in PLINK v1.07 [88]. Haplotype analysis was performed using Haploview 4.2. [92] LD blocks were defined using the method described by Gabriel *et al* [93]. Allelic association analysis of each haplotype was done using a one-sided chi-square test.

For evaluation of POAG-associated SNPs, Fisher’s exact test was performed under an allelic model. Power analysis was performed using R version 2.12.1. Association plots are shown in the figures, where r^2^ was calculated for Japanese in Tokyo and Chinese in Beijing in HapMap Phase II.

## Supporting Information

Figure S1 **Quantile-quantile plots of 531,009 SNPs in first screening data.** X-axis indicates expected p-values and Y-axis indicates observed p-values. Obtained genomic inflation factor = 1.046.
(TIF)

Figure S2 **Association plots of genotyped and imputed SNPs in the dense association mapping region.** Genotyped SNPs are shown in square and imputed SNPs in circle. The colors of the dots represent the extent of linkage disequilibrium with rs643319 (a purple diamond). Although in the screening stages, we used trend test, here allelic p-values of genotyped and imputed SNPs in the dense association mapping region spanning 87-kbp are depicted. Genes and those structures are also depicted in the lower part.
(TIF)

Table S1 **Clinical manifestations of NTG cases in each set.**
(DOC)

Table S2 **Summarized results of second screening of top 30 SNPs.**
(DOC)

Table S3 **Results of dense association mapping.**
(DOC)

Table S4 **Data from first screening of SNPs on POAG-associated loci.**
(DOC)

Table S5 **Results for 6 SNPs previously reported as candidate SNPs for POAG.**
(DOC)
