# Supplementary figures and images for: Common Variants on Chromosome 9p21 Are Associated with Normal Tension Glaucoma
Source: PLoS One. 2012 Jul 5;7(7):e40107. doi: 10.1371/journal.pone.0040107 (PMC3390321; doi:10.1371/journal.pone.0040107)

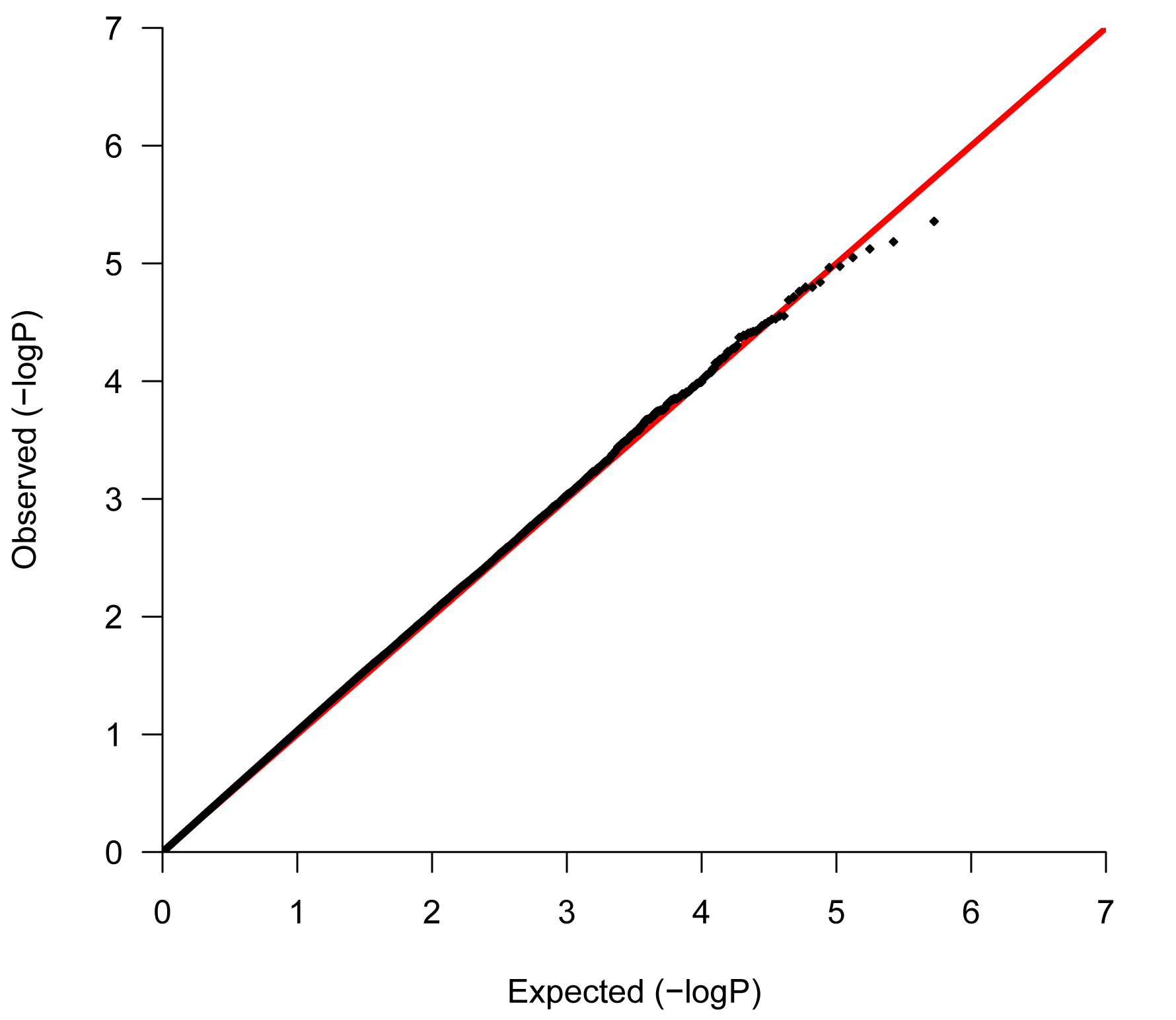

Supplement: Figure S1 — Quantile-quantile plots of 531,009 SNPs in first screening data. X-axis indicates expected p-values and Y-axis indicates observed p-values. Obtained genomic inflation factor = 1.046. (TIF) [file pone.0040107.s001.tif]

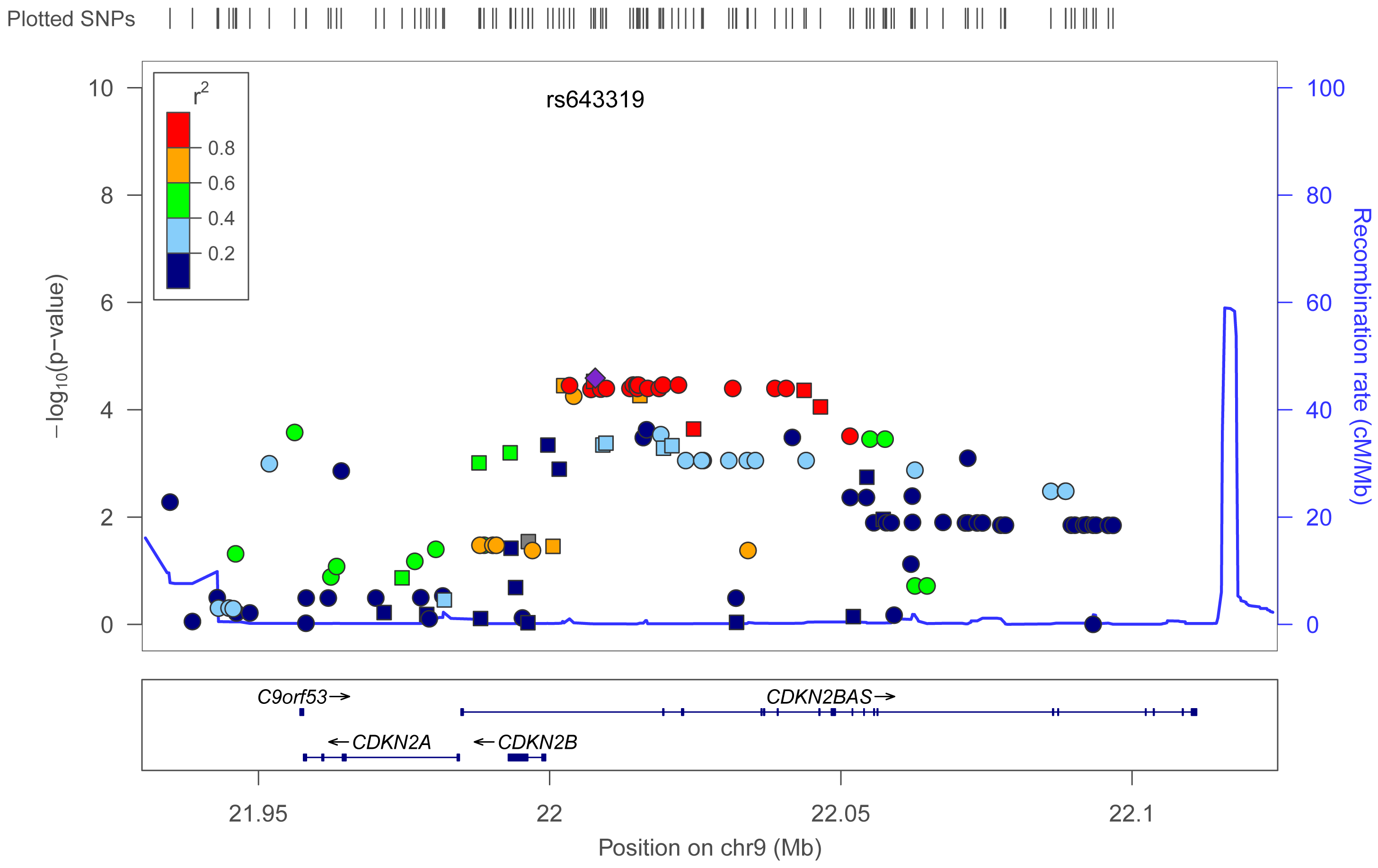

Supplement: Figure S2 — Association plots of genotyped and imputed SNPs in the dense association mapping region. Genotyped SNPs are shown in square and imputed SNPs in circle. The colors of the dots represent the extent of linkage disequilibrium with rs643319 (a purple diamond). Although in the screening stages, we used trend test, here allelic p-values of genotyped and imputed SNPs in the dense association mapping region spanning 87-kbp are depicted. Genes and those structures are also depicted in the lower part. (TIF) [file pone.0040107.s002.tif]
