# Supplementary material for: Common Variants on Chromosome 9p21 Are Associated with Normal Tension Glaucoma
Source: PLoS One. 2012 Jul 5;7(7):e40107. doi: 10.1371/journal.pone.0040107 (PMC3390321; doi:10.1371/journal.pone.0040107)
Supplement: Table S1 — Clinical manifestations of NTG cases in each set. (DOC) [file pone.0040107.s003.doc]

**Table S1. Clinical manifestations of NTG cases in each set**

|  | Age^a^ | %Male | Age at diagnosis^a^ | Refractive error^a^ | Untreated IOP^a^ | MD of HFA^a^ | Positive family history of glaucoma | Systemic diseases | | |
| --- | --- | --- | --- | --- | --- | --- | --- | --- | --- | --- |
|  |  |  |  | (Diopter) | (mmHg) |  |  | DM | Heart diseases | HT |
| Discovery cohort | 57.3±11.2 | 40.6% | 48.6±9.9 | -3.3±2.8 | 15.2±2.3 | -11.8±6.8 | 41.4% | 3.1% | 3.8% | 16.8% |
| Second screening set | 54.9±12.7 | 46.9% | 47.8±11.8 | -3.3±2.9 | 15.5±2.2 | -10.6±6.8 | 30.8% | 7.3% | 6.1% | 15.1% |
| Replication cohort | 62.2±11.6 | 36.7% | 55.3±10.6 | -2.2±3 | 15.0±2.6 | -11.3±7 | 27.7% | 4.7% | 4.0% | 27.3% |

Ophthalmologic data are from most severe eye with visual field defect.

a: Mean±SD

IOP: intraocular pressure, MD: mean deviation, HFA: Humphrey Field Analyzer, DM: diabetes mellitus, HT: hypertension
