# Supplementary material for: Common Variants on Chromosome 9p21 Are Associated with Normal Tension Glaucoma
Source: PLoS One. 2012 Jul 5;7(7):e40107. doi: 10.1371/journal.pone.0040107 (PMC3390321; doi:10.1371/journal.pone.0040107)
Supplement: Table S2 — Summarized results of second screening of top 30 SNPs. (DOC) [file pone.0040107.s004.doc]

**Table S2. Summarized results of second screening of top 30 SNPs**

| SNP | Chr | Allele | First screening | | | | | Second screening | | | | | | | Combined p-value |
| --- | --- | --- | --- | --- | --- | --- | --- | --- | --- | --- | --- | --- | --- | --- | --- |
|  |  |  | Allele frequency | | P-value | OR | (95% CI) | Assay | HW-p in ctrl | Allele frequency | | P-value | OR | (95% CI) |  |
|  |  |  | Control | Case |  |  |  |  |  | Control | Case |  |  |  |  |
| rs1298854 | 5 | C | 0.052 | 0.114 | 4.39  × 10^−6^ | 2.33 | (1.61-3.37) | TM | 1.00 | 0.097 | 0.098 | 0.979 | 1.01 | (0.67-1.51) | 0.00200 |
| rs1859999 | 17 | G | 0.355 | 0.469 | 6.56 × 10^−6^ | 1.60 | (1.30-1.96) | DT | 0.855 | 0.408 | 0.404 | 0.893 | 0.98 | (0.77-1.25) | - |
| rs4497633 | 15 | T | 0.096 | 0.171 | 7.53 × 10^−6^ | 1.95 | (1.45-2.61) | DT | 0.696 | 0.131 | 0.109 | 0.285 | 0.81 | (0.56-1.19) | - |
| rs12402031 | 1 | T | 0.435 | 0.551 | 8.91 × 10^−6^ | 1.59 | (1.30-1.96) | DT | 0.210 | 0.449 | 0.467 | 0.542 | 1.08 | (0.85-1.37) | 0.000122 |
| rs2619601 | 10 | T | 0.163 | 0.252 | 1.09 × 10^−5^ | 1.73 | (1.36-2.22) | DT | 0.0557 | 0.184 | 0.219 | 0.148 | 1.24 | (0.93-1.67) | 0.0000189 |
| **rs523096** | **9** | **A** | **0.825** | **0.904** | **1.59 × 10^−5^** | **1.99** | **(1.45-2.74)** | **DT** | **0.855** | **0.859** | **0.929** | **0.000455** | **2.15** | **(1.39-3.32)** | **7.40 × 10^−8^** |
| rs6577057 | 13 | G | 0.715 | 0.811 | 2.05 × 10^−5^ | 1.71 | (1.33-2.19) | TM | 0.155 | 0.752 | 0.692 | 0.0243 | 0.74 | (0.57-0.96) | - |
| rs6665343 | 1 | G | 0.542 | 0.649 | 2.96 × 10^−5^ | 1.56 | (1.26-1.92) | DT | 0.857 | 0.576 | 0.565 | 0.713 | 0.96 | (0.75-1.22) | - |
| rs915071 | 14 | T | 0.81 | 0.889 | 3.13 × 10^−5^ | 1.88 | (1.39-2.55) | DT | 0.495 | 0.796 | 0.809 | 0.610 | 1.08 | (0.80-1.46) | 0.000313 |
| rs2726139 | 4 | T | 0.048 | 0.101 | 3.60 × 10^−5^ | 2.22 | (1.51-3.26) | TM | 0.769 | 0.083 | 0.044 | 0.0159 | 0.51 | (0.30-0.89) | - |
| rs10831619 | 11 | G | 0.898 | 0.956 | 3.75 × 10^−5^ | 2.49 | (1.59-3.88) | DT | 0.130 | 0.883 | 0.877 | 0.752 | 0.94 | (0.65-1.36) | - |
| rs3763851 | 11 | A | 0.406 | 0.511 | 4.08 × 10^−5^ | 1.53 | (1.25-1.87) | DT | 1.00 | 0.413 | 0.396 | 0.586 | 0.93 | (0.73-1.19) | - |
| rs4868825 | 5 | T | 0.575 | 0.678 | 4.08 × 10^−5^ | 1.56 | (1.26-1.92) | DT | 0.633 | 0.639 | 0.612 | 0.356 | 0.89 | (0.70-1.14) | - |
| rs1592800 | 5 | A | 0.487 | 0.593 | 4.24 × 10^−5^ | 1.53 | (1.25-1.88) | DT | 0.285 | 0.547 | 0.538 | 0.781 | 0.97 | (0.76-1.23) | - |
| rs1585552 | 11 | T | 0.725 | 0.816 | 4.25 × 10^−5^ | 1.68 | (1.31-2.15) | DT | 0.634 | 0.752 | 0.724 | 0.297 | 0.87 | (0.66-1.14) | - |
| rs17096725 | 14 | C | 0.792 | 0.872 | 5.27 × 10^−5^ | 1.79 | (1.35-2.38) | DT | 0.424 | 0.837 | 0.825 | 0.614 | 0.92 | (0.67-1.26) | - |
| rs4142787 | 3 | T | 0.700 | 0.792 | 5.61 × 10^−5^ | 1.63 | (1.28-2.08) | DT | 0.107 | 0.740 | 0.714 | 0.346 | 0.88 | (0.67-1.15) | - |
| rs7126893 | 11 | C | 0.418 | 0.521 | 5.97 × 10^−5^ | 1.51 | (1.24-1.85) | DT | 0.0160 | 0.447 | 0.437 | 0.758 | 0.96 | (0.76-1.22) | - |
| rs9884172 | 4 | A | 0.838 | 0.909 | 6.30 × 10^−5^ | 1.93 | (1.39-2.68) | DT | 0.710 | 0.862 | 0.853 | 0.657 | 0.93 | (0.66-1.30) | - |
| rs11663168 | 18 | A | 0.101 | 0.168 | 6.95 × 10^−5^ | 1.80 | (1.35-2.42) | DT | 0.0936 | 0.137 | 0.123 | 0.514 | 0.89 | (0.62-1.27) | - |
| rs2392943 | 8 | C | 0.640 | 0.736 | 7.00 × 10^−5^ | 1.57 | (1.26-1.96) | DT | 0.757 | 0.692 | 0.694 | 0.944 | 1.01 | (0.78-1.31) | 0.00273 |
| rs11166371 | 1 | T | 0.601 | 0.699 | 7.78 × 10^−5^ | 1.54 | (1.24-1.91) | DT | 0.401 | 0.619 | 0.579 | 0.184 | 0.85 | (0.67-1.08) | - |
| rs11599868 | 10 | A | 0.822 | 0.895 | 8.81 × 10^−5^ | 1.84 | (1.35-2.51) | DT | 0.868 | 0.843 | 0.825 | 0.423 | 0.88 | (0.64-1.21) | - |
| rs10875047 | 1 | C | 0.343 | 0.441 | 8.89 × 10^−5^ | 1.51 | (1.23-1.86) | DT | 0.292 | 0.359 | 0.331 | 0.329 | 0.88 | (0.69-1.14) | - |
| rs133890 | 22 | G | 0.811 | 0.885 | 1.03 × 10^−4^ | 1.79 | (1.33-2.41) | DT | 0.649 | 0.824 | 0.806 | 0.444 | 0.89 | (0.65-1.20) | - |
| rs17114246 | 1 | G | 0.215 | 0.301 | 1.04 × 10^−4^ | 1.57 | (1.25-1.98) | DT | 0.588 | 0.204 | 0.202 | 0.932 | 0.99 | (0.73-1.33) | - |
| rs6867187 | 5 | T | 0.382 | 0.479 | 1.19 × 10^−4^ | 1.49 | (1.22-1.83) | DT | 0.786 | 0.418 | 0.407 | 0.717 | 0.96 | (0.75-1.22) | - |
| rs13167191 | 5 | A | 0.774 | 0.853 | 1.23 × 10^−4^ | 1.69 | (1.29-2.22) | DT | 0.900 | 0.776 | 0.787 | 0.662 | 1.07 | (0.80-1.43) | 0.00103 |
| rs12480536 | 20 | G | 0.070 | 0.126 | 1.43 × 10^−4^ | 1.91 | (1.36-2.68) | DT | 0.437 | 0.095 | 0.101 | 0.715 | 1.08 | (0.72-1.61) | 0.00245 |
| rs10504538 | 8 | G | 0.048 | 0.096 | 1.64 × 10^−4^ | 2.09 | (1.41-3.08) | DT | 0.673 | 0.057 | 0.058 | 0.936 | 1.02 | (0.61-1.71) | 0.00214 |

Results of first screening were obtained using Affymetrix genome-wide human SNP array 6.0.

Chr: chromosome, HW-p: p-value of Hardy-Weinberg equilibrium, DT: DigiTag2 assay, TM: TaqMan assay, OR: odds ratio, 95% CI: 95% confidence interval.
