# Supplementary material for: Common Variants on Chromosome 9p21 Are Associated with Normal Tension Glaucoma
Source: PLoS One. 2012 Jul 5;7(7):e40107. doi: 10.1371/journal.pone.0040107 (PMC3390321; doi:10.1371/journal.pone.0040107)
Supplement: Table S3 — Results of dense association mapping. (DOC) [file pone.0040107.s005.doc]

**Table S3. Results of dense association mapping**

| No. | Assay | SNP | Position (build 36) | HW-p in control | Allele | Risk allele | Allele frequency | | P-value | OR | (95%CI) | P-value in first screening | Combined p-value |
| --- | --- | --- | --- | --- | --- | --- | --- | --- | --- | --- | --- | --- | --- |
|  |  |  |  |  |  |  | Control | Case |  |  |  |  |  |
| 1 | DT | rs4074785 | 21971583 | 1.00 | A/G | A | 0.087 | 0.096 | 0.601 | 1.12 | (0.74-1.68) |  |  |
| 2 | DT | rs3731217 | 21974661 | 0.893 | C/A | A | 0.792 | 0.828 | 0.136 | 1.27 | (0.93-1.73) |  |  |
| 3 | DT | rs3731201 | 21978896 | 1.00 | C/T | T | 0.992 | 0.995 | 0.652 | 1.43 | (0.30-6.75) | 0.808 | 0.921 |
| 4 | DT | rs2811710 | 21981923 | 0.587 | T/C | C | 0.718 | 0.743 | 0.351 | 1.14 | (0.87-1.49) | 0.771 | 0.315 |
| 5 | DT | rs3218020 | 21987872 | 0.213 | G/A | A | 0.521 | 0.622 | 9.79×10^-4^ | 1.52 | (1.18-1.94) |  |  |
| 6 | DT | rs3218018 | 21988139 | 1.00 | G/T | T | 0.993 | 0.995 | 0.777 | 1.26 | (0.26-6.07) |  |  |
| 7 | DT | rs3217992 | 21993223 | 0.132 | C/T | T | 0.515 | 0.619 | 6.35×10^-4^ | 1.53 | (1.20-1.96) | 1.76×10^-4^ | 2.75×10^-7^ |
| 8 | TM | rs1063192 | 21993367 | 0.211 | G/A | A | 0.802 | 0.851 | 0.0381 | 1.41 | (1.02-1.96) |  |  |
| 9 | DT | rs2285329 | 21994153 | 0.688 | G/A | A | 0.942 | 0.959 | 0.206 | 1.45 | (0.81-2.59) |  |  |
| 10 | TM | rs2069426 | 21996273 | 1.00 | T/G | G | 0.994 | 0.995 | 0.930 | 1.07 | (0.22-5.35) |  |  |
| 11 | TM | rs974336 | 21996348 | 0.799 | T/C | C | 0.776 | 0.831 | 0.0286 | 1.42 | (1.04-1.93) |  |  |
| **12** | **TM** | **rs2069418** | **21999698** | **1.00** | **G/C** | **C** | **0.865** | **0.934** | **4.55×10^-4^** | **2.20** | **(1.40-3.46)** |  |  |
| 13 | TM | rs3808845 | 22000575 | 0.696 | A/G | G | 0.783 | 0.835 | 0.0351 | 1.41 | (1.02-1.93) |  |  |
| **14** | **DT** | **rs573687** | **22001642** | **1.00** | **A/G** | **G** | **0.862** | **0.926** | **1.28×10^-3^** | **2.00** | **(1.30-3.08)** |  |  |
| 15 | DT | rs545226 | 22002422 | 0.786 | A/G | G | 0.575 | 0.698 | 3.56×10^-5^ | 1.71 | (1.32-2.21) |  |  |
| 16 | DT | rs2106119 | 22007550 | 0.340 | A/G | G | 0.637 | 0.757 | 2.96×10^-5^ | 1.77 | (1.35-2.32) |  |  |
| 17 | DT | rs643319 | 22007836 | 0.342 | A/C | C | 0.636 | 0.757 | 2.57×10^-5^ | 1.78 | (1.36-2.33) | 3.57×10^-4^ | 5.44×10^-8^ |
| **18** | **DT** | **rs523096** | **22009129** | **0.855** | **G/A** | **A** | **0.859** | **0.929** | **4.55×10^-4^** | **2.15** | **(1.39-3.32)** | **1.59×10^-5^** | **7.40×10^-8^** |
| **19** | **DT** | **rs518394** | **22009673** | **1.00** | **C/G** | **G** | **0.858** | **0.929** | **4.19×10^-4^** | **2.16** | **(1.40-3.34)** | **1.59×10^-5^** | **6.71×10^-8^** |
| 20 | DT | rs10738604 | 22015493 | 0.928 | G/A | A | 0.574 | 0.694 | 5.45×10^-5^ | 1.68 | (1.31-2.17) |  |  |
| **21** | **DT** | **rs564398** | **22019547** | **0.855** | **C/T** | **T** | **0.859** | **0.929** | **5.21×10^-4^** | **2.13** | **(1.38-3.30)** | **2.99×10^-5^** | **1.45×10^-7^** |
| **22** | **DT** | **rs7865618** | **22021005** | **0.855** | **G/A** | **A** | **0.859** | **0.929** | **4.68×10^-4^** | **2.14** | **(1.39-3.32)** | **1.92×10^-6^** | **8.85×10^-8^** |
| 23 | DT | rs2151280 | 22024719 | 0.925 | G/A | A | 0.623 | 0.730 | 2.29×10^-4^ | 1.64 | (1.26-2.13) |  |  |
| 24 | DT | rs12352425 | 22032086 | 0.712 | A/G | A | 0.061 | 0.063 | 0.915 | 1.03 | (0.63-1.68) | 0.880 | 0.971 |
| 25 | DT | rs10965219 | 22043687 | 0.334 | A/G | G | 0.647 | 0.764 | 4.35×10^-5^ | 1.76 | (1.34-2.32) | 9.41×10^-5^ | 2.38×10^-8^ |
| 26 | DT | rs10120688 | 22046499 | 0.443 | G/A | A | 0.642 | 0.754 | 8.85×10^-5^ | 1.71 | (1.31-2.24) | 8.62×10^-5^ | 3.57×10^-8^ |
| 27 | DT | rs1011970 | 22052134 | 1.00 | T/G | T | 0.058 | 0.063 | 0.716 | 1.1 | (0.67-1.80) |  |  |
| 28 | DT | rs8181047 | 22054465 | 0.760 | A/G | G | 0.824 | 0.893 | 1.81×10^-3^ | 1.79 | (1.24-2.59) | 5.59×10^-3^ | 9.28×10^-5^ |
| 29 | DT | rs10965224 | 22057276 | 0.377 | T/A | A | 0.725 | 0.792 | 0.0110 | 1.45 | (1.09-1.93) | 0.0130 | 5.93×10^-4^ |

Bold type indicates SNPs of r^2^=1 with rs523096 in HapMap JPT.

DT: DigiTag2 assay, TM: TaqMan assay, HW-p: p-value of Hardy-Weinberg equilibrium
