# Supplementary material for: Common Variants on Chromosome 9p21 Are Associated with Normal Tension Glaucoma
Source: PLoS One. 2012 Jul 5;7(7):e40107. doi: 10.1371/journal.pone.0040107 (PMC3390321; doi:10.1371/journal.pone.0040107)
Supplement: Table S4 — Data from first screening of SNPs on POAG-associated loci. (DOC) [file pone.0040107.s006.doc]

| SNP | Chromosome | Allele | Allele frequency | | P-value | OR | (95% CI) | Relationship with POAG-associated SNP |
| --- | --- | --- | --- | --- | --- | --- | --- | --- |
|  |  |  | Control | Case |  |  |  |  |
| rs4657477 | 1 | C | 0.00360 | 0.00175 | 0.667 | 0.484 | (0.0540-4.34) | r^2^=1 with rs4656461 and rs7518099 on TMCO1 in HapMap JPT |
| rs17588172 | 7 | G | 0.219 | 0.184 | 0.0988 | 0.802 | (0.621-1.03) | r^2^=1 with rs1052990 on CAV1 and CAV2 in HapMap JPT |
| rs6969706 | 7 | T | 0 | 0.00175 | 0.340 | NA | | r^2^=1 with rs4236601 on CAV1 and CAV2 in HapMap CHB |

**Table S4. Data from first screening of SNPs on POAG-associated loci**

OR: odds ratio, NA: not assessed
