# Supplementary material for: Common Variants on Chromosome 9p21 Are Associated with Normal Tension Glaucoma
Source: PLoS One. 2012 Jul 5;7(7):e40107. doi: 10.1371/journal.pone.0040107 (PMC3390321; doi:10.1371/journal.pone.0040107)
Supplement: Table S5 — Results for 6 SNPs previously reported as candidate SNPs for POAG. (DOC) [file pone.0040107.s007.doc]

**Table S5. Results for 6 SNPs previously reported as candidate SNPs for POAG**

| SNP | Chromosome | Minor allele frequency^a^ | | P-value | OR | (95%CI) | Statistical Power^b^ |
| --- | --- | --- | --- | --- | --- | --- | --- |
|  |  | Control | Case |  |  |  |  |
| rs547984 | 1 | 0.493 | 0.526 | 0.194 | 1.14 | (0.935-1.40) | 0.810 |
| rs540782 | 1 | 0.493 | 0.526 | 0.193 | 1.14 | (0.935-1.40) | 0.810 |
| rs693421 | 1 | 0.498 | 0.528 | 0.246 | 1.13 | (0.921-1.38) | 0.828 |
| rs2499601 | 1 | 0.497 | 0.530 | 0.208 | 1.14 | (0.930-1.39) | 0.789 |
| rs7081455 | 10 | 0.236 | 0.227 | 0.685 | 0.952 | (0.749-1.21) | 0.933 |
| rs7961953 | 12 | 0.276 | 0.280 | 0.857 | 1.02 | (0.815-1.28) | 0.809 |

a: Minor alleles in controls are shown. b: alpha = 0.05
